# Dr. John C. Gilman

**Published:** 1858-01

**Authors:** 


OBITUARY.—It is with sincere regret that we announce the death of Dr. JOHN C. GILMAN, of Worcester, Mass. He graduated with high honors at the Baltimore College of Dental Surgery in 1845, and practiced his profession during the next two succeeding years in Marietta, Ohio, but at the expiration of this time he moved to Westboro, Mass., where he soon obtained an excellent practice. His partner, Dr. Tourtellatte, in a letter to the senior editor, states, "He was with me only a short time, but what I saw of him and his operations, fully warrants me in saying he was an excellent dentist and a consistent and exemplary christian—loved and respected by all who knew him. He died after a short but severe illness of acute gastritis, Sept. 11th, 1857.

Dr. Gilman spent about eighteen months of his studentship, previously to his graduation in Baltimore, and his untiring application to his professional studies, and correct and gentlemanly deportment while here, won for him the respect and friendship of all with whom he became acquainted.

